# Polypus of the Nose and Ear

**Published:** 1847-06

**Authors:** J. Robinson

**Affiliations:** European Correspondent.


### Collectanea.

*Collections from British Medical Journals—for the American Journal of Dental Science.* By J. Robinson, European Correspondent.

*Polypus of the Nose and Ear.*—Professor Velpeau in a chemical lecture delivered at the *Hospital de la Charite,* observes, you have lately seen in the wards, a case which was operated upon and in which surgical interference was rendered very difficult from the presence, at the same time, of fibrous polypi in both nasal cavities and in the pharynx. The impossibility of excision and ligature obliged us to have recourse to another method, that of crushing, which we performed in the nose; after a few days the polypus of the pharynx was spontaneously detached. To this case we now call your attention: it is important, because it might serve to establish a new treatment for polypus. In the throat, extirpation is a difficult and dangerous operation, which may be followed by hæmorrhage, a severe accident in that region. The method we employed in the case just referred to—crushing—might be likewise applied to uterine polypus. When the latter is crushed it is also cured, gangrene bringing on its separation from the womb. This is also the case when the tumors occupy the nose: in a word crushing is a safer plan for the patient and a more convenient method for the surgeon. This of course refers only to fibrous polypus.

We also have in the wards a case of vegetations of the left ear. In this region polypi are not very common; they deserve besides, to attract some degree of attention because they are usually symptomatic of another disease, and are more frequently connected with an affection of the cavity of the tympanum than of the membrana tympani. They may be cellular, fibrous or cancerous. The first form, the cellular polypus, depends often upon caries of the petrous bone, and its prognosis is of course extremely serious. In this respect a great difference must be made between the polypus of the nose and that of the ear. We do not find it produced by caries in the nasal cavities, whereas this alteration often occasions vegetations from the meatus auditorius, and is followed with abscess which sooner or later terminates in cerebral disorder. The woman who presents polypus of the ear, has always complained of hardness of hearing; sharp pains have been felt in the organ, and a discharge has for some time existed. At present, a greyish production may be distinctly seen in the ear, and its surface is free from any trace of suppuration or hæmorrhage, a fact which may lead us to admit, with some reserve, that the origin of the disease lies in the membrane, and not in caries of the bone. Long dissertations have been written on the subject of aural polypi; the ear is a region which has given rise to a family of special pathologists, who have found it advantageous to their own purposes to write long books on trifles and to say many useless things. In the ear almost all tumors, whatever their nature, assume the shape of polypi; this circumstance is clearly due to the form of the duct which, communicating with the pharynx by the eustachian tube, present besides a mucous membrane, nerves and vessels, and also with the mastoidan cells and labyrinth, is occupied by tumors as different in nature as they are in origin. When caries is suspected, it is at first useless and imprudent to examine the parts with a probe; when the instrument can penetrate into the cavity of the tympanum, it may lacerate one of the three delicate little muscles which are attached to its bones, and, coming into contact with these, may furnish, on account of the extreme thinness of their periosteum, an erroneous sensation which could readily be mistaken for that given by caries and if that sign were consulted, a false diagnosis might be the consequence.

